# *In Vivo* Antiplasmodial Activity of *Entandrophragma cylindricum* (Sprague) Sprague Ethyl Acetate Extract in *Plasmodium berghei*-Infected Mice

**DOI:** 10.1155/2020/8846067

**Published:** 2020-11-13

**Authors:** Noumedem Anangmo Christelle Nadia, Yamssi Cédric, Ngongang Ouankou Christian, Simeni Njonnou Sylvain Raoul, Yondo Jeanette, Mounvera Abdel Azizi, Sop Foka Eric Igor, Djape Guangue Diane, Chahdini Gbambie Abass, Tsila Henri Gabriel, Mpoame Mbida

**Affiliations:** ^1^Department of Microbiology, Haematology and Immunology Faculty of Medicine and Pharmaceutical Sciences, University of Dschang, P.O. Box 96, Dschang, Cameroon; ^2^Department of Biomedical Sciences, Faculty of Health Sciences, University of Bamenda, P.O. Box 39 Bambili, Cameroon; ^3^Department of Internal Medicine and Specialties, Faculty of Medicine and Pharmaceutical Sciences, University of Dschang, P.O. Box 96, Dschang, Cameroon; ^4^Department of Animal Biology, Faculty of Science, University of Dschang, P.O. Box 067, Dschang, Cameroon

## Abstract

**Background:**

One of the most dangerous Plasmodium species is *Plasmodium falciparum*. Hence, it causes a higher rate of mortality. The resistance of *Plasmodium falciparum* to the ACT (Artemisinin-based Combination Therapies) has led to the search for new antimalarial drugs. The purpose of this research was to assess the *in vivo* antiplasmodial activity of *Entandrophragma cylindricum* ethyl acetate extract to provide a scientific basis for the use of this medicinal plant to treat malaria.

**Methods:**

*Entandrophragma cylindricum* stem bark powder was macerated in ethyl acetate to obtain the extract. The extract liquid filtrate was concentrated, evaporated and dry using a Rotavapor. The Peter and Rane test were used for the suppressive and curative antiplasmodial activities at different doses (125, 250 and 500 mg/kg). A positive and negative control groups were administered chloroquine (5 mg/kg) and 10% hypromelose, respectively. To assess the parasitemia of the mice a thin blood smear was made.

**Results:**

The ethyl acetate extract completely (100%) inhibited the development of *P. berghei* in the suppressive test at the dose of 500 mg/kg while that of the curative test was inhibited at 95%. The extract-treated group (500 mg/kg) and (Chloroquine (5 mg/kg) group all survived. The negative control group recorded a 100% mortality rate.

**Conclusion:**

The present study provides scientific confirmation on the use of *E. cylindricum* stem bark as an antiplasmodial remedy. However, the identification of the mode of action and the purification of the active compounds are necessary for further studies.

## 1. Background

Malaria is a parasitic disease caused by protozoa called Plasmodium which is transmitted to humans by the bite of an infected female anopheles mosquito. One of the main public health problems in developing countries is malaria. According to the World Health Organization [1], in 2017, they were an estimate of 251 million cases of malaria as compared to 231 million cases in 2010. Cameroon with 71% of the total population living in high transmission areas and 4500 deaths recorded annually is one of the most affected countries in Africa [2]. The control of malaria has been a multi-faceted approach involving the control of the vector and the use of drugs for treatment [3]. Currently, artemisinin-based combination therapies (ACTs) are the major drugs for malaria treatment [1]. However, the cost of ACTs is high [4]. Unfortunately, there are reports of increasing drug resistance to artemisinin and its derivatives [5]. The emergence of multi-drug resistant strains of Plasmodium exacerbates the situation further; posing a major obstacle to successful chemoprophylaxis and chemotherapy of the disease [6].

Since many modern drugs such as artemisinin and quinine originate from plants, it is essential that other medicinal plants that have a folklore reputation for antimalarial properties are explored, to recognize their safety and efficacy and to determine their potential as sources of new antimalarial drugs [6].

*Entandrophragma cylindricum* belonging to the family Meliaceae is a medicinal plant traditionally used in the Centre Region of Cameroon to treat malaria, yellow fever, bacterial infections like typhoid fever, diarrhea and symptoms like stomach-ache. The bioassay-guided fractionation of the stem bark of *Entandrophragma cylindricum* Sprague carried out by Kouam et al. [7] reveals the presence of four acyclic triterpene derivatives named sapelenins G–J (1–4), along with eight known compounds, sapelenins A–D, ekeberin D2 (5), (+)-catechin and epicatechin, and anderolide G. One of the most promising sources of compounds with antiplasmodial properties has been studied in the plant family Meliaceae. Some species belonging to this family (*Azadirachta indica, Entandrophragma angolense, Entandrophragma candollei, Entandrophragma utile, Khaya grandifoliola*) are commonly used as antimalarial agents [8, 9].

Similarly, Noumedem et al. [10] reported a high *in vitro* activity of *Entandrophragma cylindricum* against *P. falciparum* and a non-cytotoxicity against L929 cells.

An extract can be active *in vitro* and inactive *in vivo* due to some biochemical processes in the host organism [11]. It is therefore imperative to assess extracts *in vivo* antiplasmodial activity to confirm their activities. The overall aim of this research was to assess the *in vivo* antimalarial efficacy of *E. cylindricum* ethyl acetate extract to justify its usage by traditional healers and ascertain its potential as an antiplasmodial drug.

## 2. Material and Methods

### 2.1. Plant Material

Stem barks, leaves, flowers and fruit of *Entandrophragma cylindricum* were harvested in Makenene, situated in Mbam Inoubou, Division, Centre Region of Cameroon, in March 2015. The authentication was done by, Mister Victor Nana. The number (No 1716/SRFCam) was given as the voucher specimen.

### 2.2. Preparation of Ethyl Acetate Extract

Ethyl Acetate extract was obtained using the procedure described by Wabo Poné et al. [12]. Ethyl acetate solvent was used to prepare the extract by maceration using 100 g of stored powder for 72 hours. A rotary vapor (BUCHI R-210) under vacuum (40°C) was used to obtain the extract. The ethyl acetate extract yield was calculated using the formula below.

(1) $$\begin{array}{c} \text{Yield}=\frac{\text{Mass}\text{of}\text{extract}}{\text{Mass}\text{of}\text{dry}\text{plant}\text{powder}}*100 \end{array}$$

The extract was stored for further processing.

#### 2.2.1. Reference Drugs

The reference drugs, Chloroquine (CQ) was obtained from SIGMA and were used as positive controls for *Plasmodium berghei* (ANKA). Ten percent (10%) hypromelose was used as negative controls.

### 2.3. *In vivo* Pharmacological Studies

#### 2.3.1. Preparation of the Parasite Inoculum

*Plasmodium berghei* (ANKA) was used to infect mice intraperitoneally by using 0.2 mL of infected blood in isotonic saline containing 5 x 10^7^*P.berghei* erythrocyte per milliliter.

#### 2.3.2. The Peter's 4-Day Suppressive Test

The Peter's 4-day suppressive test [13] was used, with thirty-six (36) mice (female and male) of average weight 20 g infected by intraperitoneal inoculations of 5 x 10^7^*P. berghei* infected erythrocytes per milliliter. The first three groups of 6 mice each were administered 125, 250 and 500 mg/kg. The positive and negative controls were Chloroquine (5 mg/kg) and 10% hypromelose, respectively. The 6^th^ group was non-infected and non-treated and was considered as the neutral control group. These products were administered daily, orally, during 4 consecutive days, to infected mice. A thin blood smear was made on the last day. The suppression test was evaluated as follows:

(2) $$\begin{array}{c} \text{Average}\text{suppression}\text{of}\text{parasitemia}=\frac{\%\text{parasitemia}\text{control}–\%\text{parasitemia}\text{of}treatmentgroup}{\%\text{parasitemia}\text{control}} \end{array}$$

The mean survival time for 29 days (D0-D28), was calculated as follows: (No. of days survived)/(total No. of days (29) ×100.

#### 2.3.3. Evaluation of Curative Activity

The curative activity was evaluated using Ryley and Peter's method [14]. On the first day, 30 mice (male and female) were infected intravenously with 1x10^7^*P. berghei* parasitized erythrocytes. Chloroquine (5 mg) (n =6) and 10% Hypromelose (n =6) were used as positive and negative control, respectively. The third group (n =6) received 500 mg/kg of crude extract. The 4^th^ group (n =6) was non-infected and non-treated and was considered as the neutral control group. The mice were treated once daily for 7 days and the parasitemia monitor by the May-Grünwald-Giemsa staining technique after 72 h of infection. The mean survival time was determined as in the suppressive test for 30 d (D0–D29). The mean reduction rate was calculated as: % reduction =100 × [(C – T)/C], Where T and C represent the average parasitemia of the treated and control group.

### 2.4. Phytochemical Screening

The ethyl acetate extract was tested for the presence of phenolic compounds, alkaloids, flavonoids, Polyphenols, tannins, saponin, triterpenes and steroids using standard procedures described by Builders et al. [15].

### 2.5. Statistical Analysis

ANOVA (One- way) followed by a post-test (Turkey-Kramer multiple comparison tests) was used to analyze the data. Differences between means were considered significant at 5% level of significance that is p ≤0.05.

## 3. Results

### 3.1. Plant Extract Yields

The yields obtained after extraction of 100 g of stored powder of *E. cylindricum* with ethyl acetate solvent was 10.11%.

### 3.2. Suppressive Antiplasmodial Activity

**Figure 1:**
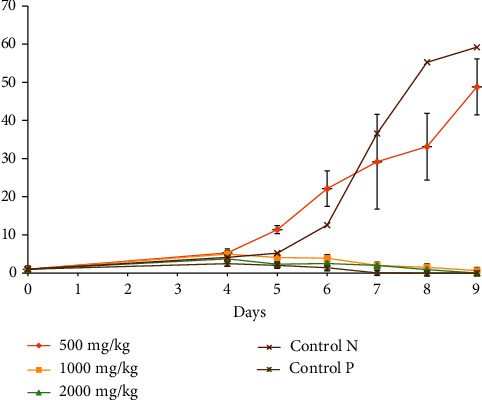
Effect of *E. cylindricum* on parasitemia.

It follows from the analysis of Figure 1 that the oral administration of 125 mg/kg body weight did not influence parasitemia as compared to the reference drug and doses (250, and 500 mg/kg) which totally inhibited the growth of the parasite.

It follows from the analysis of Table 1 that 500 mg/kg and Chloroquine had a 100% survival rate during the 30 days post-treatment. A low survival rate (9.16 ± 1.04) was observed for the 10% Hypromelose treated group.

### 3.3. Effect of *E. cylindricum* Extract on Hematological Parameters

Table 2 presents the effect of crude extract on hematological parameters. It appears from this table that, hematological analysis (Table 3) showed no significant (*P* >0.05) changes in the RBC and WBC of treated mice.

HCT: Hematocrit, HGB: Hemoglobin, MCHC: Mean corpuscular hemoglobin concentration, MCV: Mean corpuscular volume, PL: Platelets. DIRBC: Distribution index of red blood cells, MPV: Mean platelet volume.

### 3.4. Curative Activity

**Figure 2:**
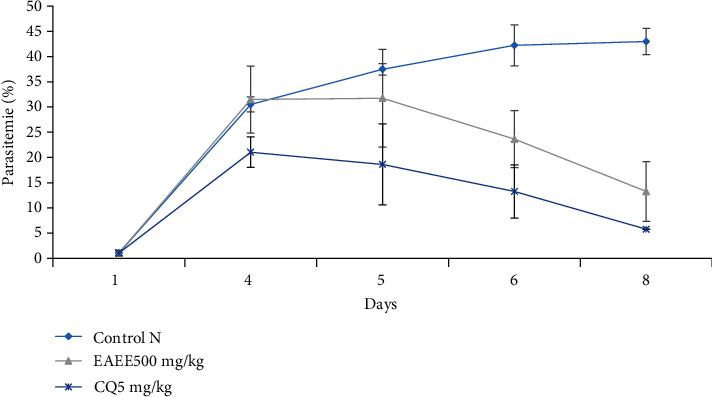
Curative effect of *E. cylindricum* crude extract.

It follows from the analysis of Figure 2 that, the extract treated group parasitemia on day7 and 8 was 23.64%, and 13.25%, respectively. Infection in the negative and positive control groups was 43.01% and 5.75%, respectively, on the 8^th^ day.

### 3.5. Cumulative Effect and Mortality Rate of Experimental Animals after Treatment

Table 3 shows the cumulative number (n) and the mortality rates (%) of experimental animals after treatment with *E. cylindricum* extract during the curative test. It appears from this table that, from the 15^th^ day they was a mortality rate of 33.33% still the 30^th^ day of the experiment. All the animals in the Chloroquine (5 mg/kg) group survived during the ‘experiment. The negative control group recorded a 100% mortality rate.

### 3.6. Phytochemical Analysis

Table 4 presents the phytochemical screening of the ethyl acetate extract. It follows from the analysis of this Table that all the chemical groups were present (alkaloids,

flavonoids, saponines, terpenoids and tannins,) except steroids.

## 4. Discussion

The exponential increase in the number of parasites in the blood of mice during the 4^th^ day of infection testifies the establishment of the infection [13].

The level of parasitemia is reduced in a dose-dependent manner which is an indicator of the antiplasmodial activity of the plant. According to Adugna et al. [16] and Trigg and Kondrachine [17], an extract is consider effective if it can have more than 30% suppression of the parasitemia as compare to the control group, which is in agreement with this study findings. This study demonstrates that *E. cylindricum* ethyl acetate extract has very high and dose-dependent chemosuppression. The same observations were made by Léa et al. [18] whereby extracts of *Guiera senegalensis* and *Bauhinia rufescens* leaves produced a significant reduction of parasitemia.

Crude extract of *Entandrophragma cylindricum* can stimulate some enzymes involved in the fight against oxidative stress hence causing less damage to the host [10]. The anemia observed in the infected untreated group indicates the destruction or hemolysis of the red blood cells [19, 20].

One of the main indicators used to evaluate the antimalarial activity of plant extract is the mean survival time [13]. No mouse died in the extract-treated group (500 mg/kg) and Chloroquine (5 mg/kg). Our results show that the extract enhances the survival time of mice at all doses levels which are directly linked to parasitemia suppression. Akuodor *et al.* [21] made similar observations when studying *Bombax buonopozense* against *Plasmodium berghei.* The survival of these animals treated may be due to the active compounds. According to Noumedem *et al.* [10], the crude extract of *Entandrophragma cylindricum* can stimulate some enzymes involved in the fight against oxidative stress hence causing less damage to the host.

On day 8^th^ of the curative test, the crude extract did not completely inhibit the parasite even though we had a suppressive effect. Akuodor *et al.* [21] obtained similar results. These authors showed that parasites are less vulnerable to the suppressive test than to the curative test when they assess *Bombax buonopozense* root bark aqueous extract in mice infected by *Plasmodium berghei*. This difference in inhibition may be because the treatment was administered 1 h (suppressive test) and 72 h (curative test) after infection. It is evident that *E. cylindricum* have antiplasmodial activities.

As a preliminary phase to look for compounds that potentially have antiplasmodial activity, phytochemical screening was performed. The phytochemical screening reveals the presence of secondary metabolites (alkaloids, flavonoids, Saponines, Terpenoids and Tannins,) except steroids. The antiplasmodial activity of this plant may be due to these secondary metabolites in the plant extract. It has been found that certain alkaloids possess antimalarial properties. A key example is the isolation of quinine from the Cinchona species. Their antiplasmodial activity may be through the inhibition and detoxification of heame in red blood cells [22].

## 5. Conclusion

The resistance of *P. falciparum* to chloroquine is now a major health problem in some African countries. The results provide scientific data that, the extract of *E. cylindricum* may contain antimalarial active compounds hence justifies their use as antimalarial agents in Cameroon. The development of phytopharmaceutical products could be of relevance for the protection of risk groups, in areas with such high levels of resistance. Therefore, it would be interesting if the active principle is isolated, identified, and characterized.

## Figures and Tables

**Table 1:** Suppressive effect, Parasitemia level and Mean survival rate.

Treatment	Dosage(mg/kg)	Parasitemia level	% of suppression	Mean survival rate
Hypromelose 10%	0	59.24 ± 4.72	/	9.16 ± 1.04
Chloroquine	5	0 ± 0	100 ± 0	30 ± 0
Extracts	125	48.83 ± 2.3	17.57 ± 2.62	11.5 ± 3.8
	250	0.63 ± 0.02	98.93 ± 0.01	27 ± 1.3
	500	0 ± 0	100 ± 0	30 ± 0

**Table 2:** Effect of treatment on hematological parameters.

pH treatments	Doses (mg/kg)	WBC (x 10^9^/I)	RBC (x 10^12^/I)	Pl(%)	MPV(fl)	PDW	HGB (g/dl)	HCT(%)	MCV(fl)	MCHC(Pg)	MCHC(g/dl)	DIRBC(%)
Neutral control		7.6 ±1.1∗	9.10 ± 0.5∗	0.45±0.05	6.60±0.20	14.20±0.10	14.3±1.60∗	48.3 ± 0.8∗	54.22±3.40	15.70±0.70	31.10±0.20	17.70±0.50
Negative control	0	14.10±2.10	6.80±0.40	0.32±0.07	6.90±0.20	14.50±0.20	8.70±1.20	32.2±6.50	53.30±3.20	14.80±0.40	29.10±1.20	18.10±0.10
CQ	5	9.10 ± 0.60∗	8.20 ± 0.43∗	0.39±0.06	6.70±0.10	14.10±0.20	10.60 ± 1.20∗	44.10 ± 0.40∗	54.10±2.40	15.00±0.40	31.40±0.30	17.90±0.20
	125	9.89 ± 0.85∗	7.62 ± 0.08∗	0.32±0.13	7.21±0.03	15.02±0.16	9.79 ± 0.13∗	43.03 ± 0.62∗	51.33±0.11	14.96±0.46	29.78±0.17	18.12±0.13
Crude extract	250	10.32 ± 0.11∗	8.25±0.22	0.36±0.21	6.81±0.10	14.18±0.40	10.34 ± 0.08∗	41.63 ± 1.79∗	51.79±0.23	14.64±0.22	30.06±0.41	17.31±0.09
	500	10.32 ± 0.13∗	8.28 ± 0.29∗	0.34±0.08	6.66±0.16	14.14±0.21	10.31 ± 0.32∗	43.01 ± 1.21∗	53.17±0.65	14.99±0.31	31.02±0.21	17.42±0.45

**Table 3:** Cumulative number (n) and mortality rate (%) of the experimental animals after treatment.

Treatment	Doses(mg/kg)	POST TREATMENT PERIOD (DAY) n (%)				
		10	15	20	25	30
Hypromellose 10%	0	6 (100)	6 (100)	6 (100)	6 (100)	6 (100)
Chloroquine	5	0 (0)	0 (0)	0 (0)	0 (0)	0 (0)
*E. cylindricum*	500	1 (16.67)	2 (33.33)	2 (33.33)	2 (33.33)	2 (33.33)

**Table 4:** Phytochemical screening of *Entandrophragma cylindricum* ethyl acetate extract.

Chemical groups	Ethyl acetate extract
Alkaloids	+
Flavonoids	+
Polyphenols	+
Tannins	+
Saponins	+
Steroids	—
Terpenoids	+

+ = present, - = absent.

## Data Availability

Data and material are available to other researchers upon request.
